# Comparison of clinical characteristics and disease burden of febrile seizures in children with and without COVID-19

**DOI:** 10.1186/s12887-024-04821-z

**Published:** 2024-05-13

**Authors:** Zhongli Jiang, Cuiyun Fang, Fengyimei Peng, Wei Fan

**Affiliations:** 1 Department of Pediatrics, Liyang People’s Hospital, Liyang, China; 2 Department of Nursing, Liyang People’s Hospital, Liyang, China

**Keywords:** Febrile seizures, COVID-19, Clinical characteristics, Disease burden, Children

## Abstract

**Background:**

Febrile seizures (FS) are the most common seizure disorder in children and a common neurologic complication in children with coronavirus disease 2019 (COVID-19). This study aimed to identify differences in clinical characteristics and disease burden between FS with and without COVID-19.

**Materials and methods:**

We conducted a retrospective analysis of medical data at our hospital from December 2019 to July 2023, focusing on hospitalized patients under the age of 14 diagnosed with FS who underwent COVID-19 polymerase chain reaction (PCR) testing. Descriptive statistics and analysis of variance were employed to compare the COVID-19 and non-COVID-19 groups in terms of clinical characteristics and disease burden.

**Results:**

A total of 514 patients were included, with 106 testing positive for COVID-19 and 408 testing negative. Patients with COVID-19 were older (34.87 ± 6.16 vs. 28.61 ± 11.35 months, *P* < 0.001) and had a higher proportion of males (79.2% vs. 62.3%, *P* = 0.001). The COVID-19 group had longer seizure durations (4.57 ± 4.38 vs. 3.22 ± 2.91 min, *P* = 0.006) and more complex FS (25.5% vs. 15.9%, *P* = 0.022). Laboratory tests showed lower lymphocyte counts in the COVID-19 group (1.87 ± 1.48 vs. 2.75 ± 1.51 × 103/µL, *P* < 0.001) and higher creatine kinase levels (158.49 ± 82.89 vs. 110.89 ± 56.11 U/L, *P* < 0.001). No significant differences were found in hospital costs, length of hospitalization, and intensive care unit admissions.

**Conclusion:**

Clinicians should be knowledgeable about the distinct clinical characteristics of FS in children with COVID-19. Despite distinct features, the prognosis remains favorable and does not require excessive intervention. Ongoing monitoring and research are needed to fully understand the impact of COVID-19 on FS and optimize management strategies.

## Introduction

Febrile seizures (FS) are the most prevalent seizure disorder in children, ranging from 2 to 5% in the United States and Western Europe, and higher prevalence rates of 5-10% in India and 6-9% in Japan among Asian populations [1]. FS are characterized by a seizure accompanied by a fever (temperature ≥ 100.4 °F) and do not involve central nervous system infection [2]. They typically occur in children aged 6 months to 5 years and can be categorized as simple or complex FS. Simple FS are primary generalized seizures that last for less than 15 min and do not recur within 24 h [3]. They occur during fevers unrelated to acute neurologic illness. On the other hand, complex febrile convulsions are defined as focal or prolonged seizures lasting ≥ 15 min and/or recurring within 24 h. These seizures may be linked to postictal neurologic abnormalities, commonly known as Todd’s palsy, or may occur in children with pre-existing neurologic deficits. This category also includes children whose seizures stopped before the 15th minute due to the administration of anti-seizure medication [4]. The exact etiology of FS is not fully understood, but it is believed to be multifactorial, involving genetics, viral infections, certain vaccinations, and incomplete neurological development in children [5]. Common viruses associated with FS include human herpesvirus 6, influenza virus, adenovirus, parainfluenza virus, varicella virus, respiratory syncytial virus, and rotavirus [6]. Despite having different seasonal distributions, the characteristics of seizures caused by different respiratory viruses do not show significant differences [7].

Coronavirus disease 2019 (COVID-19) is a severe infectious disease caused by the severe acute respiratory syndrome coronavirus 2 (SARS-CoV-2), with the epidemic starting in December 2019. In addition to respiratory abnormalities, COVID-19 also affects the nervous system and leads to a variety of complications [8]. Based on a multicenter prospective observational study, the most common neurological complications among children hospitalized for COVID-19 were fatigue, myalgia, altered consciousness, seizures, anosmia, and dysgeusia, in descending order of prevalence [9]. FS have become a common neurologic manifestation in children with COVID-19, particularly during the Omicron epidemic [10]. A multicenter cross-sectional study in the United States reported a 3.9% prevalence of FS with COVID-19 in hospitalized children [11]. While many studies have reported the clinical characteristics of FS in the context of COVID-19 at different time points, there is limited literature focusing on potential differences in clinical characteristics and disease burden between FS with and without COVID-19 [12, 13]. Therefore, the aim of this study is to compare the demographic characteristics, clinical features, laboratory findings, and disease burden of febrile seizures in children with and without COVID-19, with the intention of contributing to this area of research.

## Materials and methods

### Study design

We conducted a comprehensive search of the electronic medical record system at Liyang People’s Hospital, the sole tertiary hospital in the region, to retrieve the complete medical records of inpatients from December 2019 to July 2023. Two experienced pediatric specialists independently reviewed the medical records. Patients who met the diagnostic criteria for FS and underwent COVID-19 polymerase chain reaction (PCR) testing were included in the study. Exclusion criteria included central nervous system infections, prior history of seizures, metabolic disorders, head trauma, drug-induced seizures, incomplete medical records and those unable to obtain informed consent. In cases where a patient had multiple hospitalizations during the study period, only the information from the last medical record was collected.

We collected demographic data, which included age, sex, history of FS, and family history of FS. Clinical characteristics and laboratory test results were also gathered. Moreover, cerebrospinal fluid tests, electroencephalography, and brain computed tomography results were included in the data collection. Additionally, information regarding the disease burden, such as hospital costs, length of hospital stays, and admission to the intensive care unit, was documented. The calculation of hospital costs takes into consideration various factors such as the cost of medical supplies, equipment, staffing, overhead expenses, and administrative costs.

FS are characterized by a seizure accompanied by a fever (temperature ≥ 100.4 °F) and do not involve central nervous system infection [2]. Simple FS are primary generalized seizures that last for less than 15 min and do not recur within 24 h [3]. Complex febrile convulsions are defined as focal or prolonged seizures lasting ≥ 15 min and/or recurring within 24 h [3]. Status epilepticus was defined as a single sustained seizure lasting more than 5 min, or frequent clinical seizures without return to baseline clinical status in the interictal period [14]. COVID-19 PCR testing was conducted by a qualified laboratory that adheres to national standards [15]. The time lapse between febrile seizures and the COVID-19 PCR testing averaged around 0.5–3 h. Based on the results of the COVID-19 PCR tests, patients were divided into COVID-19 and non-COVID-19 groups. A case-control study unmatched was then conducted to analyze the differences between the two groups.

The study was conducted in accordance with the Declaration of Helsinki, and approved by the Institutional Review Board of Liyang People’s Hospital (protocol code YJ2023012 and date of approval February 15, 2023). Informed consent was obtained from the participants’ legal guardian/next of kin.

### Statistical analysis

Categorical variables were presented as n (%) and compared using appropriate statistical tests, such as chi-square tests, Fisher’s exact tests, or continuity corrections. Continuous variables were expressed as mean ± standard deviation. The significance of normally distributed data was analyzed using the t-test, while the significance of non-normally distributed data was analyzed using the Wilcoxon rank sum test. Statistical significance was defined as a two-sided P-value of less than 0.05. All statistical analyses in our study were performed using SPSS software (version 20.0; SPSS, Chicago, Illinois, United States of America).

## Results

### General information and demographic data

**Fig. 1:**
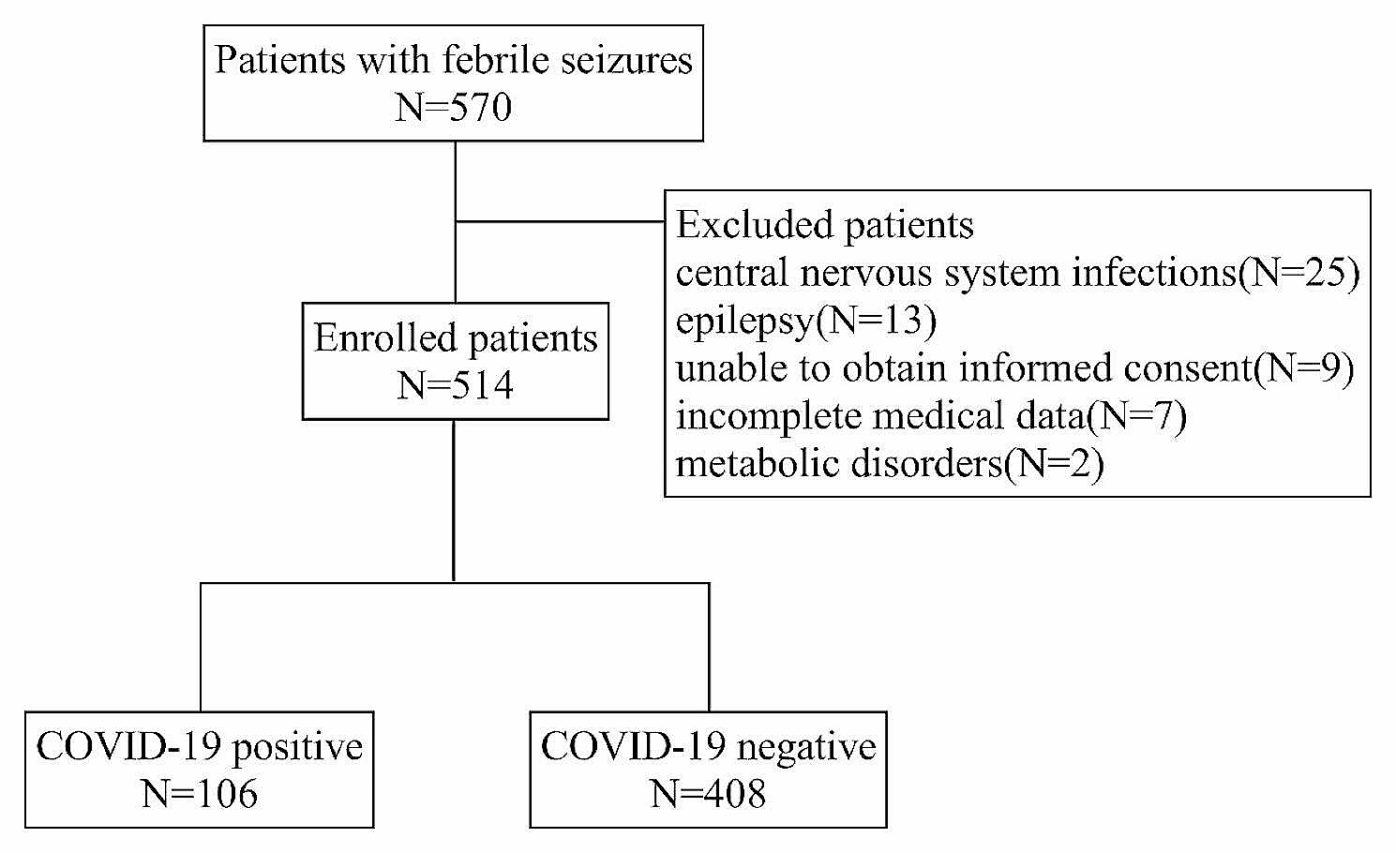
Flow chart for patient inclusion and exclusion

During the study period, a total of 570 patients were diagnosed with FS and underwent COVID-19 PCR testing. A total of 56 patients were excluded from our study, including central nervous system infections (25 patients), epilepsy (13 patients), unable to provide informed consent (9 patients), incomplete medical data (7 patients), and metabolic disorders (2 patients). A total of 514 patients were included in this study. The flow of patient inclusion and exclusion was shown in Fig. 1. Of these patients, 338 (65.8%) were male and 176 (34.2%) were female. The age of onset of FS ranged from 6 to 58 months, with a mean age of 29.9 ± 10.79 months. A history of FS was present in 166 (32.3%) patients, and 22 (4.3%) patients had a family history of FS.

The interval between febrile seizures and the COVID-19 PCR testing was approximately 0.5–3 h. COVID-19 PCR testing was positive in 106 (20.6%) patients and negative in 408 (79.4%) patients. Table 1 summarizes the demographic data of the two patient groups. Patients with COVID-19 were older compared to those without COVID-19 infection (34.87 ± 6.16 vs. 28.61 ± 11.35 months, *P* < 0.001). Additionally, there was a higher proportion of males in the COVID-19 group (79.2% vs. 62.3%, *P* = 0.001). There was no significant difference in the history of FS and family history of FS between the two groups.

### Clinical characteristics

The highest recorded temperature during the course of the disease in all patients ranged from 38 °C to 41 °C, with a mean of 39.33 ± 0.50 °C. The duration of fever ranged from 1 to 5 days, with a mean duration of 2.37 ± 0.95 days. The most common type of seizure was generalized tonic-clonic seizure in 494 (96.1%) patients. Thirty-eight (7.4%) patients experienced seizure recurrences within 24 h. The mean duration of seizures was 3.49 ± 3.31 min. Fifty-four (10.6%) patients presented with status epilepticus and 92 patients were categorized as having complex FS. A total of 51 (10.0%) patients required antiseizure medications.

As shown in Table 1, compared with FS without COVID-19, patients with COVID-19 had a significantly longer duration of seizures (4.57 ± 4.38 vs. 3.22 ± 2.91 min, *P* = 0.006) and a significantly higher proportion of complex FS (25.5% vs. 15.9%, *P* = 0.022). No significant differences were observed between the two groups in terms of fever peak, duration of fever, interval between seizures and fever, type of seizures, recurrence of seizures within 24 h, status epilepticus, and antiseizure medication needs.

**Table 1:** The demographic data and clinical characteristics of the study patients

	COVID-19 (*n* = 106)	NON-COVID-19 (*n* = 408)	*P*	OR (95%CI)
Sex, male	84 (79.2%)	254 (62.3%)	0.001*	0.427 (0.241–0.756)
Age (months)	34.87 ± 6.16	28.61 ± 11.35	< 0.001*	1.063 (1.038–1.089)
History of FS	37 (31.4%)	118 (28.9%)	0.232	2.052 (1.236–3.408)
Family history of FS	6 (5.7%)	16 (3.9%)	0.604	1.018 (0.305–3.399)
Fever peak (℃)	39.39 ± 0.54	39.32 ± 0.48	0.225	2.104 (1.223–3.622)
Fever duration (d)	2.38 ± 0.90	2.37 ± 0.97	0.925	0.813 (0.702–0.942)
Interval between fever and seizures (h)	8.62 ± 7.30	10.18 ± 9.41	0.068	0.975 (0.943–1.007)
Type of seizures, GTC	100 (94.3%)	394 (96.6%)	0.290	0.796 (0.137–4.625)
Duration of seizures (min)	4.57 ± 4.38	3.22 ± 2.91	0.006*	1.032 (0.930–1.144)
Recurrent seizures within 24 h	12 (11.3%)	26 (6.4%)	0.083	1.460 (0.211–7.121)
Status epilepticus	16 (15.1%)	38 (9.3%)	0.084	3.432 (0.380–9.004)
Complex FS	27 (25.5%)	65 (15.9%)	0.022*	2.611 (0.403–6.931)
Antiseizure medications need	14 (13.2%)	37 (9.1%)	0.204	0.482(0.198–1.172)

Data are presented as number (%) or mean ± SD values. *SD* standard; *COVID-19* coronavirus 2019; *OR* odds ratio; *CI* confidence intervals; *FS* febrile seizures; *GTC* generalized tonic-clonic

*Significant difference (*P* < 0.05) was noted between the groups

### Laboratory tests and brain tests

In laboratory tests, the COVID-19 group had significantly lower lymphocyte counts compared to the non-COVID-19 group (1.87 ± 1.48 vs. 2.75 ± 1.51 × 10^3^/µL, *P* < 0.001). Additionally, creatine kinase levels were significantly higher in the COVID-19 group compared to the control group (158.49 ± 82.89 vs. 110.89 ± 56.11 U/L, *P* < 0.001). There were no significant differences in terms of neutrophil count, hemoglobin, platelets, C-reactive protein, procalcitonin, serum sodium, serum potassium, aspartate aminotransferase, and alanine aminotransferase. The laboratory results are summarized in Table 2.

A total of 49 patients underwent electroencephalography in the acute phase, with 17 in the COVID-19 group and 32 in the non-COVID-19 group. Among them, 9 patients in both groups showed background slow wave enhancement, which normalized during the outpatient review after 1 month. Sixteen patients underwent brain computed tomography, and no abnormalities were detected. Three patients underwent cerebrospinal fluid examination, and the results showed no abnormalities.

**Table 2:** The Laboratory tests and disease burden of the study patients

	COVID-19 (*n* = 106)	NON-COVID-19 (*n* = 408)	*P*	OR (95%CI)
White blood cells (×10^3^/µL)	8.15 ± 4.47	8.87 ± 3.19	0.121	0.584 (0.402–0.849)
Neutrophil (×10^3^/µL)	5.61 ± 3.80	4.92 ± 3.36	0.068	1.697 (1.176–2.450)
Lymphocyte (×10^3^/µL)	1.87 ± 1.48	2.75 ± 1.51	< 0.001*	1.153 (0.732–1.816)
Hemoglobin (g/L)	125.11 ± 8.21	123.5 ± 14.02	0.128	1.006 (0.987–1.024)
Platelets (×10^3^/µL)	231.75 ± 100.02	250.03 ± 67.15	0.077	0.998 (0.994–1.002)
C-reactive protein (mg/dL)	5.19 ± 1.40	5.01 ± 2.33	0.814	1.010(0.974–1.048)
Procalcitonin (ng/mL)	1.03 ± 0.58	1.02 ± 0.56	0.845	1.064 (0.807–1.402)
Sodium (mmol/L)	137.42 ± 2.77	202.29 ± 37.09	0.476	1.000 (0.996–1.003)
Potassium (mmol/L)	4.42 ± 0.43	4.33 ± 0.38	0.053	1.974 (1.002–3.890)
Creatine kinase (U/L)	158.49 ± 82.89	110.89 ± 56.11	< 0.001*	1.009 (1.005–1.013)
Aspartate aminotransferase (U/L)	42.30 ± 12.02	41.26 ± 12.00	0.423	0.976 (0.950–1.003)
Alanine aminotransferase (U/L)	19.96 ± 6.81	21.61 ± 15.36	0.102	1.007 (0.979–1.035)
Hospital costs (Chinese Yuan)	1974.44 ± 495.75	2058.72 ± 525.85	0.125	0.906 (0.743–1.105)
Length of hospitalization (d)	4.43 ± 1.44	4.53 ± 1.50	0.567	1.025 (0.852–1.234)
ICU admission	6 (5.7%)	11 (2.7%)	0.134	4.446 (1.735–11.393)

Data are presented as mean ± SD values. *SD* standard; *ICU* intensive care unit; *OR* odds ratio; *CI* confidence intervals

*Significant difference (*P* < 0.05) was noted between the groups

### Disease burden

The average hospital costs were 2041.34 ± 520.43 yuan, and the average length of hospitalization was 4.51 ± 1.48 days. Additionally, 17 (3.3%) patients received critical care services. No patients died or required chronic antiseizure medications. There were no significant differences between the COVID-19 and non-COVID-19 groups in terms of hospital costs, length of hospitalization, and proportion of intensive care unit admissions, as shown in Table 2.

## Discussion

The objective of our study was to compare the clinical characteristics and disease burden of FS in children with and without COVID-19. Our study yielded several noteworthy findings. Firstly, FS associated with COVID-19 occurred at an older age and had a significantly higher prevalence among males. Secondly, these seizures had a longer duration and a higher incidence of complex FS. Lastly, FS with COVID-19 were associated with lower lymphocyte counts and higher creatine kinase levels. In terms of disease burden, there were no disparities in hospital costs, length of hospitalization, and proportion of intensive care unit admissions between FS with and without COVID-19.

Since the start of the pandemic in 2020, the neurological impact of COVID-19 has been a source of concern. Neurological complications in children with COVID-19 have been documented in recent international multicenter studies [9, 16]. The most common neurological complication reported was malaise, followed by altered consciousness and myalgia [9]. It is important to note that these complications can occur not only in children with pre-existing underlying conditions but also in previously healthy children [16]. Additionally, although rare, there are serious and potentially life-threatening neurological complications associated with COVID-19. A large multi-center study conducted in the United States found that 2.5% of hospitalized children and adolescents with acute COVID-19 or multisystem inflammatory syndrome in children (MIS-C) developed various life-threatening neurological disorders associated with COVID-19 [17].

Our study uncovered a significant increase in the age of onset of FS in children with COVID-19 compared to those without COVID-19, aligning with previous studies [12, 18]. This suggests that COVID-19 infection may exert distinct effects across different age groups. One plausible explanation for this pattern is the heightened severity of illness and higher fever observed in older children with COVID-19 [19]. FS are typically triggered by elevated body temperatures, and it is plausible that the more severe illness and higher fever in older children with COVID-19 may contribute to the delayed onset of FS. Another potential factor is the systemic inflammatory response and cytokine storm associated with COVID-19. Older children may possess a more mature immune system, which could result in a more robust inflammatory response to viral infections such as COVID-19 [20]. This heightened inflammatory response can impact the central nervous system and lower the seizure threshold, rendering older children more susceptible to FS. However, it is important to note that the effect of age on the severity of COVID-19 and the risk of death was significantly reduced after adjusting for important age-related risk factors such as immunocompromised conditions, prior respiratory diseases, and hypertension [21]. To comprehensively comprehend the mechanisms underpinning the age-specific effects of COVID-19 and FS, further research is imperative.

While both groups of children with FS were predominantly male, our study found a higher proportion of males in the COVID-19 group. The impact of COVID-19 on gender differences in FS has been observed in previous studies [18]. While both males and females are equally susceptible to COVID-19 infection, males tend to experience more severe complications and outcomes [22]. However, the reason for this gender difference is not well understood. One possible explanation for this finding is the potential differences in immune response between males and females. Studies have shown that females tend to mount stronger immune responses to viral infections than males, which may confer some degree of protection against FS [23, 24]. Additionally, hormonal differences between males and females may also play a role in this gender discrepancy. For example, estrogen has been shown to have a neuroprotective effect, which may explain why females are less susceptible to FS [25].

In the COVID-19 group, patients had a significantly longer duration of seizures and a significantly higher proportion of complex FS. These imply that COVID-19 has stronger neurological effects. Various neurological manifestations have been associated with COVID-19, including altered consciousness, fatigue, seizures, and changes in smell and taste [9]. The exact mechanism of neurological complications caused by COVID-19 remains unclear, but some studies have suggested that COVID-19 may affect the central nervous system through the olfactory mucosa, blood-brain barrier, and axonal transport [26, 27]. It is highly unlikely that the neurological complications in patients with COVID-19 were caused by direct infection of the central nervous system with SARS-CoV-2, as indicated by the negative PCR test results for the virus in the majority of cerebrospinal fluid tests [28]. Instead, it is believed that the inflammatory response theory, supported by blood-brain barrier dysfunction and elevated cytokine levels in patients with COVID-19, may play a role in these neurological complications [29].

The discrepancy observed in our research, where FS associated with COVID-19 exhibited significantly lower lymphocyte counts compared to FS without COVID-19, warrants further discussion. The immune response to viral infections, including COVID-19, is known to involve various immune cells, including lymphocytes. Lymphocytes play a crucial role in the adaptive immune response and are responsible for recognizing and eliminating viral pathogens [30]. The lower lymphocyte counts observed in FS with COVID-19 could be attributed to the direct impact of the virus on lymphocyte production or increased lymphocyte destruction. COVID-19 has been shown to cause lymphopenia in some patients, which may be due to the virus infecting and damaging lymphocytes or inducing their apoptosis [31]. Additionally, the systemic inflammatory response triggered by COVID-19 could lead to increased consumption and redistribution of lymphocytes to affected tissues [32]. It is important to note that our study was limited to laboratory tests and did not assess the functional capacity of lymphocytes or investigate the specific subsets of lymphocytes affected. Furthermore, our study revealed that patients with FS and COVID-19 had higher creatine kinase levels compared to those without COVID-19. Increased creatine kinase is frequently observed in COVID-19 patients and may be indicative of disease severity, serving as a predictor of poor prognosis [33, 34]. It has also been reported that creatine kinase is associated with increased levels of inflammatory factors in COVID-19 patients [35]. However, the exact mechanism behind the elevated creatine kinase in the context of COVID-19 remains unclear. It is uncertain whether this elevation is a result of a viral-induced inflammatory response or direct muscle toxicity. Further investigation is needed to elucidate the underlying mechanisms involved in the association between COVID-19 and elevated creatine kinase levels in patients with FS.

The lumbar puncture rate observed in this study was remarkably low. Several factors may account for this phenomenon. Firstly, patients diagnosed with central nervous system infections post-lumbar puncture were intentionally excluded from the study. Secondly, the cautious approach required by physicians when considering lumbar puncture in China can be attributed to the general reluctance of Chinese parents towards invasive procedures. Our criteria for recommending lumbar puncture typically encompass patients presenting with unexplained lethargy, vomiting, or positive signs of meningeal irritation and/or pathology [36, 37]; those lacking a history of influenza or pneumococcal vaccination, or with an unknown vaccination status between 6 and 12 months of age [3]; and individuals who have received antibiotic therapy, particularly those under 18 months old [2]. The decline in lumbar punctures among these patients may be associated with the increased vaccination rates and decreased antibiotic usage in China.

Despite presenting with more prolonged and complex FS, our study found that patients with FS and COVID-19 did not experience an increased burden of illness associated with FS, including length of hospitalization, hospital costs, and intensive care unit admissions. This finding suggests that while COVID-19 may contribute to the development of more severe and urgent seizures, it does not necessarily result in a greater need for extensive medical interventions or supportive measures. The majority of patients with FS and COVID-19 were able to recover without requiring additional medical interventions beyond standard febrile seizure management.

There are some limitations to our study. Firstly, it is a single-center retrospective study, which may be subject to selection bias and recall bias. Additionally, it is important to consider the timing and context of our study findings. Given that China’s anti-epidemic policy was fully liberalized during the Omicron period, caution should be exercised when extrapolating our results to all SARS-CoV-2 variants. Furthermore, our study only included patients who were admitted to the hospital, which may have excluded patients with milder symptoms who did not require hospitalization. As a result, our findings may not be applicable to children with FS who did not seek medical attention or were managed in an outpatient setting. Future studies with larger sample sizes and more diverse populations are needed to confirm our findings and provide a more comprehensive understanding of the impact of COVID-19 on FS in children.

## Conclusion

In conclusion, patients with COVID-19 were older and had a higher proportion of males compared to those without the infection. Those with COVID-19 also experienced longer seizure durations and a higher rate of complex FS. Laboratory findings showed lower lymphocyte counts and higher creatine kinase levels in the COVID-19 group. In terms of disease burden, there were no disparities in hospital costs, length of hospitalization, and proportion of intensive care unit admissions between FS with and without COVID-19. As the pandemic continues to evolve, ongoing monitoring and research are necessary to understand the full impact of COVID-19 on FS and optimize management strategies for these patients.

## Data Availability

The original contributions presented in the study are included in the article. Further inquiries can be directed to the corresponding author.

## Abbreviations

FS: Febrile seizures
COVID-19: Coronavirus disease 2019
PCR: Polymerase chain reaction
SARS-CoV-2: Severe acute respiratory syndrome coronavirus 2
SD: Standard
GTC: Generalized tonic-clonic
ICU: Intensive care unit
